# Atypical Myrosinase as a Mediator of Glucosinolate Functions in Plants

**DOI:** 10.3389/fpls.2019.01008

**Published:** 2019-08-06

**Authors:** Ryosuke Sugiyama, Masami Y. Hirai

**Affiliations:** RIKEN Center for Sustainable Resource Science, Yokohama, Japan

**Keywords:** glucosinolate, myrosinase, beta-glucosidase, metabolism, stress response

## Abstract

Glucosinolates (GLSs) are a well-known class of specialized plant metabolites, distributed mostly in the order Brassicales. A vast research field in basic and applied sciences has grown up around GLSs owing to their presence in important agricultural crops and the model plant *Arabidopsis thaliana*, and their broad range of bioactivities beneficial to human health. The major purpose of GLSs in plants has been considered their function as a chemical defense against predators. GLSs are physically separated from a specialized class of beta-thioglucosidases called myrosinases, at the tissue level or at the single-cell level. They are brought together as a consequence of tissue damage, primarily triggered by herbivores, and their interaction results in the release of toxic volatile chemicals including isothiocyanates. In addition, recent studies have suggested that plants may adopt other strategies independent of tissue disruption for initiating GLS breakdown to cope with certain biotic/abiotic stresses. This hypothesis has been further supported by the discovery of an atypical class of GLS-hydrolyzing enzymes possessing features that are distinct from those of the classical myrosinases. Nevertheless, there is only little information on the physiological importance of atypical myrosinases. In this review, we focus on the broad diversity of the beta-glucosidase subclasses containing known atypical myrosinases in *A. thaliana* to discuss the hypothesis that numerous members of these subclasses can hydrolyze GLSs to regulate their diverse functions in plants. Also, the increasingly broadening functional repertoires of known atypical/classical myrosinases are described with reference to recent findings. Assessment of independent insights gained from *A. thaliana* with respect to (1) the phenotype of mutants lacking genes in the GLS metabolic/breakdown pathways, (2) fluctuation in GLS contents/metabolism under specific conditions, and (3) the response of plants to exogenous GLSs or their hydrolytic products, will enable us to reconsider the physiological importance of GLS breakdown in particular situations, which is likely to be regulated by specific beta-glucosidases.

## Introduction

Over the years, a number of bioactive metabolites have been identified in plants, many of which are utilized as beneficial sources of pharmaceuticals and/or research tools. Glucosinolate (GLS), a class of sulfur-rich natural products mainly produced by the family Brassicaceae, is among the most studied plant metabolites owing to its potential health-related benefits and availability in the model plant *Arabidopsis thaliana* (Halkier and Gershenzon, 2006; Agerbirk and Olsen, 2012). GLSs impart specific pungency and flavors to Brassicaceae vegetables such as mustard and cabbage (Fahey et al., 2001; Halkier, 2016; Possenti et al., 2017). Moreover, a few GLS compounds such as glucoraphanin (4-methylsulfinyl-*n*-butyl glucosinolate) are known to produce health-promoting chemicals with diverse bioactivities (Traka, 2016; Banerjee and Paruthy, 2017). Very recently, the enormous body of research activities on GLS has been compiled as two books entitled *Glucosinolates* with few overlaps (Kopriva, 2016; Mérillon and Ramawat, 2017).

**FIGURE 1:**
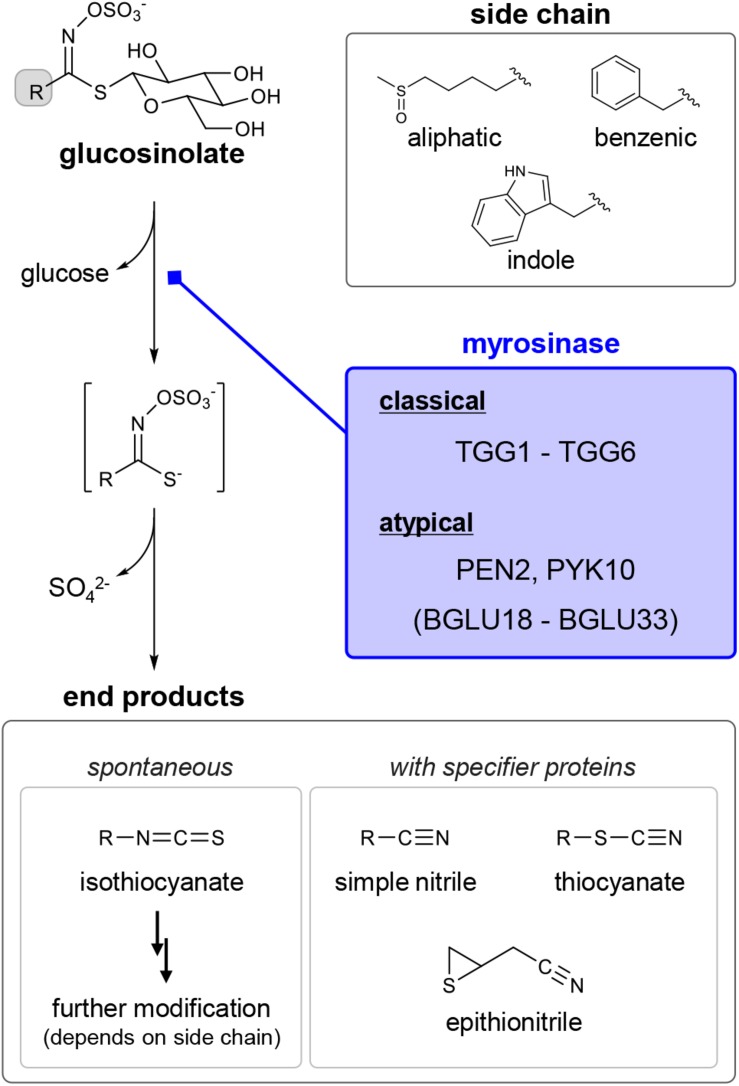
General understanding of the diversity in glucosinolates (GLSs), their breakdown products and myrosinases in *Arabidopsis thaliana*. Each subclass of GLSs — aliphatic, benzenic and indole — is biosynthesized from different precursor amino acids; in the case of *A. thaliana*, mainly methionine, phenylalanine and tryptophan, respectively. Classical and atypical myrosinases catalyze hydrolytic removal of the beta-D-thioglucoside moiety from GLS. In general, a Lossen-type rearrangement of the unstable intermediate results in the generation of isothiocyanate. Specifier proteins and side chain structures are responsible for conversion of the intermediate into other end products.

In recent years, chemical ecology, which focuses on gaining an understanding of the physiological functions of specialized metabolites (previously referred to as secondary metabolites) in organisms, has also become a burgeoning scientific field in natural product research. In this context, GLSs have traditionally been considered as defense chemicals deployed against predators. GLSs generally accumulate in specific cells (S-cells), separated from cells containing their hydrolytic enzymes (beta-thioglucosidases called myrosinases) (Halkier, 2016; Wittstock et al., 2016a). In addition, it has been suggested that GLSs and myrosinases could co-exist even within single cells, probably being compartmentalized in different organelles (Koroleva and Cramer, 2011). They are mixed upon tissue damage to release toxic volatiles such as isothiocyanates (ITCs) (Figure 1). Specifier proteins and side chain structures play an important role in converting the unstable aglycon to various end products with different bioactivities (Lambrix et al., 2001; Hanschen et al., 2014; Wittstock et al., 2016a, b; Eisenschmidt-Bönn et al., 2019). For instance, simple nitriles are generated in the presence of nitrile specifier proteins, and ITCs possessing a hydroxyl group at position 2 can be further cyclized. These compartmentalizations, which enable plants to safely control harmful chemicals, is referred to as the GLS–myrosinase system or “mustard oil bomb” and has long fascinated many plant scientists (Lüthy and Matile, 1984).

Knowledge on the chemical diversity embedded in the GLS metabolism and on the molecular mechanisms underlying the GLS–myrosinase system is frequently updated. For example, energetical investigations have been made of GLS biosynthetic genes (Halkier, 2016; Barco and Clay, 2019), end products directed by specifier proteins (Wittstock et al., 2016a), tissue localization via GLS transporters (Jørgensen et al., 2015; Halkier, 2016), differences in GLS contents among species and accessions (Kliebenstein and Cacho, 2016), and proteins that interact with myrosinases to regulate their activity and stability (Bhat and Vyas, 2019; Chen et al., 2019). However, most of the insights that have been gained regarding the molecular basis and physiological importance of GLS breakdown are based on the intercellular and tissue damage-dependent GLS–myrosinase system. On the other hand, there are several reports on fluctuations of endogenous GLS levels even in non-disrupted tissues, caused by pathogen attack or abiotic stress (Martinez-Ballesta et al., 2013; Variyar et al., 2014; Burow, 2016; Pastorczyk and Bednarek, 2016). These observations suggest the existence of different system(s) that regulate GLS breakdown independent of tissue disruption, to cope with such environmental stresses. In connection with the subcellular GLS–myrosinase compartmentalization, additional functions of GLSs not limited to their role as the defense chemicals against herbivores have been recognized (Bednarek, 2012b; Katz et al., 2015; Francisco et al., 2016b; Burow and Halkier, 2017). In fact, many of the latest studies have demonstrated broad physiological functions of GLSs (Francisco et al., 2016a; Katz and Chamovitz, 2017; Malinovsky et al., 2017; Nintemann et al., 2018; Urbancsok et al., 2018a, b).

In order to gain a more comprehensive understanding of the possible multi-functionality of GLSs *in planta*, we should take a global view of the profound diversification embedded in GLSs (Figure 1). Nearly 150 GLS compounds have been identified to date, and there are at least 36 GLSs with different side-chain structures in *A. thaliana* (Brown et al., 2003; Agerbirk et al., 2018). Three subclasses of GLSs — aliphatic, benzenic and indole GLSs — are biosynthesized from different precursor amino acids with independent regulatory systems by MYB and MYC transcription factors (Figure 1) (Frerigmann, 2016). Thus, it is conceivable that each GLS class could participate in distinct biological processes, as indole GLSs are known to play an essential role in plant immunity via coordination with the metabolism of other phytoalexins (Bednarek et al., 2009; Pedras et al., 2011; Klein and Sattely, 2015, 2017; Frerigmann et al., 2016). In addition, different bioactivities of ITCs, dependent on their side-chain structures, are well recognized despite their non-specific nucleophilicity, which may contribute to fitness performance of plants (Burow et al., 2010; Andersson et al., 2015; Urbancsok et al., 2017). Moreover, the production of various end products even from a single GLS species, directed by the specifier proteins, is likely to expand the endogenous biological targets of GLSs in different signaling pathways (Lambrix et al., 2001; Wittstock et al., 2016a, b). In contrast, the genetic and biochemical diversity in myrosinases is less understood, even though a new class of beta-glucosidases capable of hydrolyzing GLSs has been identified. Compared with the well-documented class of myrosinases that are widely found in the order Brassicales, these so-called atypical myrosinases possess unique features with respect to both amino acid sequences and enzymatic profiles. In this review, we therefore focus on the considerable diversity of beta-glucosidases in *A. thaliana* as a model to discuss their possible contribution to the broad utility of GLSs in plants, even at the subcellular level.

## Classical Versus Atypical Myrosinases

In *A. thaliana*, glucosyl hydrolase family I is composed of 47 *BETA-GLUCOSIDASE* (*BGLU*) genes and a *BGLU*-like gene, *AFR2* (Xu et al., 2004). According to the crystal structure of a thioglucosidase from *Sinapis alba*, a few amino acid residues are conserved in myrosinases among a wide range of GLS-producing plants (Burmeister et al., 1997). For example, Gln and Glu residues within the catalytic site are considered to be essential for cleavage of the thioglucoside moiety. Thus, six genes (*BGLU34*–*BGLU39*) named *THIOGLUCOSIDE GLUCOHYDROLASE* (*TGG*) had previously been considered as the only class encoding myrosinases in *A. thaliana*. Myrosinases possessing these amino acid signatures have been found in a wide range of GLS-producing plants (Rask et al., 2000).

In 2009, however, it was revealed that PENETRATION 2 (PEN2)/BGLU26 is capable of hydrolyzing indole GLSs and that generation of the putative degradation products is critical for the plant immune response (Bednarek et al., 2009; Clay et al., 2009). Although the key Gln residue is replaced by Glu in PEN2, the recombinant PEN2 protein clearly showed myrosinase activity against indol-3-ylmethyl glucosinolate (I3G) and its 4-methoxy analog (Bednarek et al., 2009). Moreover, Nakano et al. (2014) demonstrated that PYK10/BGLU23 is a major component of the endoplasmatic reticulum (ER) body — an organelle found primarily in the family Brassicaceae — and also has functions as a myrosinase against I3G (Nakano et al., 2017). PYK10 also has Glu instead of the key Gln residue found in TGGs. Based on these findings, PEN2 and PYK10 were newly categorized as atypical or EE-type myrosinases, in contrast to TGG1–TGG6, which are referred to as classical or QE-type myrosinases (“EE” and “QE” represent their conserved amino acid residues).

**FIGURE 2:**
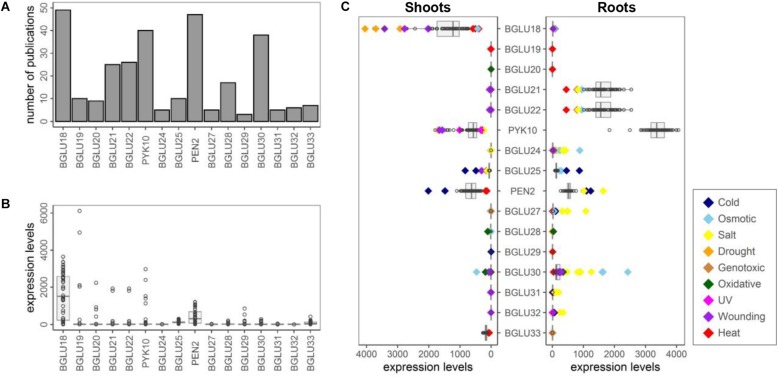
Expression patterns of *BGLU18*–*BGLU33* in *Arabidopsis thaliana*. Publications and Affymetrix ATH1 array data were extracted using ePlant (https://bar.utoronto.ca/eplant/). In the ATH1 chip, *BGLU21* and *BGLU22* are crosshybridized to the same probe. **(A)** The number of publications by 2017 extracted using each BGLU as a query. **(B)** Expression levels of *BGLU18*–*BGLU33* in different tissues. Original data comes from Nakabayashi et al. (2005) and Schmid et al. (2005). Top three tissues exhibiting the highest signal levels of each gene are summarized in Supplementary Table S1. **(C)** Expression levels of *BGLU18*–*BGLU33* in shoots and roots under different abiotic stresses for 0, 0.25, 0.5, 1, 3, 4, 6, 12, and 24 h. Original data comes from Kilian et al. (2007). Dots exhibiting more than 2-fold or less than 1/2-fold expression of control are highlighted as colored diamonds. Cold (navy), continuous 4°C on crushed ice in cold chamber; Osmotic (skyblue), 300 mM mannitol; Salt (yellow), 150 mM NaCl; Drought (orange), rafts were exposed to the air stream for 15 min with loss of app.10% fresh weight; Genotoxic (brown), bleomycin 1.5 μg/ml plus mitomycin C 22 μg/ml; Oxidative (green), 10 μM methyl viologen; UV-B (magenta), 15 min UV-B light field; Wounding (purple), punctuation of the leaves by three consecutive applications of a custom made pin-tool consisting of 16 needles; Heat (red), 3 h at 38°C followed by recovery at 25°C.

It should be noted that the two Glu residues identified in PEN2 and PYK10 are conserved among the 16 genes named *BGLU18*–*BGLU33* in *A. thaliana* (Table 1). A phylogenomic analysis of BGLUs from more than 50 plant species revealed that the monophyletic clade composed of these 16 BGLUs is specific for the order Brassicales (Nakano et al., 2017). These BGLU members lack the Gln and other amino acid signatures conserved in the classical myrosinases, whereas additional basic residues oriented to the deduced substrate-binding pocket occur only in this subclass. Detailed amino acid signatures in *A. thaliana* BGLUs are shown in Nakano et al. (2017), especially in their Figure 5 and Supplementary Figure S11. Considering the distinct amino acid signatures conserved in each BGLU subclass and their frequent emergence across plant species, it is suggested that the QE and EE myrosinases have arisen independently during evolution (Nakano et al., 2017). Not limited to PEN2 and PYK10, interestingly, transcriptional changes and mutations in some of these *BGLUs* have suggested their relevance in response to specific stresses (Figure 2, Tables 1, 2, and Supplementary Tables S2, S3). It is also to be noted that GLS metabolism is affected independent of tissue disruption under those conditions (Table 2). Therefore, other members of this BGLU subclass may have myrosinase activities and regulate different machineries for GLS turnover that are perhaps more specialized in substrate selectivity, tissue localization and developmental stage, rather than the broad-scale chemical defense against herbivores deployed by classical myrosinases.

**TABLE 1:** Current insights on BGLU18–BGLU39, putative EE-type myrosinases in *Arabidopsis thaliana*.

**Class^a^**	**Gene name**	**AGI ID**	**Substrates^b^**	**Subcellular localization**	**Phenotypes of knock-down mutants**	**Notes**
III	BGLU18/BG1	At1g52400	ABA-GE^1^ **(4MI3G)**^2^	ER body^3^	Susceptible to *A. vulgare* attacks (*pyk10 bglu18*)^2^ susceptible to drought^1^	A major component of inducible ER bodies^4^
	BGLU19	At3g21370			Tolerant to salt stress^5^	Up-regulation by high NaCl^5^
	BGLU20/ATA27	At1g75940				
	BGLU21	At1g66270	Coumarin glucosides^6^	ER body^3^		A component of root ER bodies^7^ cannot hydrolyze sinigrin^6^
	BGLU22	At1g66280	Coumarin glucosides^6^	ER body^3^		A component of root ER bodies^7^ cannot hydrolyze sinigrin^6^
	BGLU23/PYK10	At3g09260	Coumarin glucosides^6^ **I3G,**^8^ **(4MI3G)**^2^	ER body^3^	Susceptible to *A. vulgare* attacks (*pyk10 bglu18*)^2^	A major component of root and leaf ER bodies^7^ cannot hydrolyze sinigrin^6^
	BGLU24	At5g28510				Very low signals in all tissues^8^
	BGLU25/GLUC	At3g03640				
IV	BGLU26/PEN2	At2g44490	**indole GLSs**^9^ 4-MUG^9^	Peroxisome^10^	Susceptible to pathogen attacks^10^	
	BGLU27	At3g60120				Very low signals in all tissues^8^
V	BGLU28	At2g44460				Up-regulation by sulfur depletion^11^
	BGLU29	At2g44470				
	BGLU30/SRG2/DIN2	At3g60140				Up-regulation by sulfur depletion,^11^ extended darkness^12^ or senescence^13^
	BGLU31	At5g24540				Very low signals in all tissues^8^
	BGLU32	At5g24550				Very low signals in all tissues^8^
VI	BGLU33/BG2	At2g32860	ABA-GE^14^	Vacuole^14^	Susceptible to salinity^14^	

*^*a*^Xu et al. (2004). ^*b*^Glucosinolates are bolded. ABE-GE, abscisic acid glucosyl ester; 4MI3G, 4-methoxyindol-3-ylmethyl glucosinolate; I3G, indol-3-ylmethyl glucosinolate; 4-MUG, 4-methylumbelliferyl-beta-D-*O*-glucoside. ^1^Lee et al. (2006), ^2^Nakazaki et al. (2019), ^3^Yamada et al. (2011), ^4^Ogasawara et al. (2009), ^5^Cao et al. (2017), ^6^Ahn et al. (2010), ^7^Matsushima et al. (2003), Nagano et al. (2008), ^8^Schmid et al. (2005), Nakabayashi et al. (2005), ^9^Bednarek et al. (2009), ^10^Lipka et al. (2005), ^11^Hirai et al. (2003, 2004), Nikiforova et al. (2003), Maruyama-Nakashita et al. (2003), ^12^Fujiki et al. (2001), ^13^Lee J. et al. (2007), ^14^Xu et al. (2012).*

**TABLE 2:** Relevance of glucosinolates (GLSs) and the Brassicales-specific beta-glucosidases (BGLUs) in *Arabidopsis thaliana* under abiotic stress.

**Stress**	**GLS levels**	**Related BGLUs**	**Involvement in GLS metabolism**	**Effects of ITC treatment^a^**
Drought	Decrease^1^	BGLU18^2^ TGG1, TGG2^3^	Guard cells accumulate a large amount of TGG1 and TGG2.^3^	AITC induces stomatal closure.^4^
Salinity	Increase^5^	BGLU19,^6^ BGLU33^7^	*myb28 myb29* is more susceptible to salt stress.^5^	–
Sulfur deficiency	Decrease^8^	BGLU28,^9^ BGLU30^9^	Growth of *gtr1 gtr2* is defected under low sulfur.^10^	–
Light/Dark	Increase by light/Decrease in the dark^11^	BGLU30^12^ TGG1, TGG2^13^	GLS biosynthetic genes shows the diurnal rhythm.^11^	–
Temperature	–	Not determined	A low-GLS mutant (TU8) is more susceptible to heat stress.^14^	AITC and PEITC enhances heat tolerance.^15^

*^*a*^AITC, allyl isothiocyanate; PEITC, phenethyl isothiocyanate. ^1^Ren et al. (2009), ^2^Lee et al. (2006), ^3^Zhao et al. (2008), Islam et al. (2009), ^4^Khokon et al. (2011), ^5^Martinez-Ballesta et al. (2015), ^6^Cao et al. (2017), ^7^Xu et al. (2012), ^8^Falk et al. (2007), ^9^Hirai et al. (2003, 2004), Maruyama-Nakashita et al. (2003), Nikiforova et al. (2003), ^10^Nour-Eldin et al. (2012), ^11^Huseby et al. (2013), ^*12*^Fujiki et al. (2001), ^13^Brandt et al. (2018), ^14^Haughn et al. (1991), Ludwig-Müller et al. (2000), ^15^Hara et al. (2012).*

In this review, we aim to discuss the hypothesis that a wide range of these Brassicales-specific BGLUs can function as myrosinases to regulate the multiple functions of GLSs *in planta*. Although current insights into this BGLU subclass are highly limited, essentially three types of previous studies could be useful for considering this hypothesis: analyses of (1) the phenotype of mutants lacking genes responsible for the GLS metabolic/breakdown pathway; (2) the changes in GLS levels/metabolism under specific conditions; and (3) the response of plants treated with GLSs or their hydrolytic products (Table 2). Here, mainly based on studies in *A. thaliana*, we consider phenotypic information related to *BGLU18*–*BGLU33*, fluctuations in GLS metabolism, and the effects of exogenous GLS breakdown products on plants to review the physiological importance of GLS breakdown in particular situations. In addition to a specific focus on atypical myrosinases, more diverse functions of classical TGGs are described with reference to the most recent insights. Finally, we suggest the types of experiment that are effective in gaining a more complete understanding of the multi-functionality of GLSs in plants.

## A Plant Immune Pathway Regulated by PEN2, the First Atypical Myrosinase

Here, we describe the general understanding of the PEN2 pathway in brief, as the relevance of PEN2 and indole GLSs in plant immunity has been well documented (Bednarek, 2012a; Johansson et al., 2014; Frerigmann et al., 2016; Pastorczyk and Bednarek, 2016; Xu et al., 2016). PEN2 was first identified as a component of the pre-invasive resistance in *A. thaliana* against the powdery mildew fungi, *Blumeria graminis* f. sp. *hordei* and *Erysiphe pisi* (Lipka et al., 2005; Bednarek et al., 2009). PEN2 is also responsible for the callose deposit in *A. thaliana* seedlings induced by fungal pathogens or flg22, a bacterial flagellin-derived peptide (Clay et al., 2009), even though it is suggested that penetration resistance and callose deposition are not directly linked (Lipka et al., 2005; Maeda et al., 2009; Hiruma et al., 2010). These stimuli induce a decrease of indole GLS levels mediated by PEN2 and the accumulation of plausible end products including indol-3-ylmethylamine (I3A), raphanusamic acid (RA), and 4-*O*-beta-D-glucosyl-indol-3-yl formamide (4OGlcI3F) (Bednarek et al., 2009, 2011; Lu et al., 2015). Notably, hydroxylation of I3G at position 4 mediated by CYP81F2 is a critical step for the PEN-dependent immune response. This is an interesting example of a GLS with a particular side chain exhibiting a specialized biological function. On the other hand, the unstable indol-3-ylmethyl ITC can serve as an intermediate for the biosynthesis of several phytoalexins (Bednarek et al., 2009; Klein and Sattely, 2017). Thus, the molecular mechanisms and actual bioactive metabolite(s) relevant in this pathway remain to be further investigated.

In a recent study, a co-expression analysis for genes involved in the PEN2 immune system identified GLUTATHIONE *S*-TRANSFERASE CLASS-TAU MEMBER 13 (GSTU13) as a critical component of that pathway (Piślewska-Bednarek et al., 2018). The *gstu13* mutants were more susceptible to several pathogens and were impaired in callose deposition induced by the bacterial flg22 epitope. Furthermore, the formation of pathogen-triggered specific metabolites such as indol-3-ylmethyl amine was broadly repressed in these mutants. Therefore, the conjugation of ITCs with glutathione catalyzed by GSTU13 was revealed to be strictly essential for the activation of the indole GLS-related immune system.

## PYK10, BGLU18 and ER-Retained BGLUS Could Maintain an Intracellular GLS–Myrosinase System

In the GLS–myrosinase system, physical separation of glucose-conjugated precursor compounds from their hydrolases is an efficient means to control the bioactivity of these metabolites, and this strategy may function even at the subcellular level. The ER body, a rod-shaped organelle continuous with the ER, is considered to provide such an intracellular compartment; a few BGLUs including PYK10/BGLU23 are significantly enriched in these structures in *A. thaliana* (Matsushima et al., 2003; Ogasawara et al., 2009). Among the Brassicales-specific BGLUs, BGLU18–BGLU25 commonly have ER-retention signals in their signal peptides and C-terminal regions (Nakano et al., 2014). Indeed, ER bodies that are constitutively present in roots accumulate large amounts of PYK10 and the closest homologs BGLU21 and BGLU22, whereas BGLU18 is the major component of another class of ER body that is induced by wounding or methyl jasmonate treatment (Matsushima et al., 2002, 2003; Nagano et al., 2008; Ogasawara et al., 2009). More detailed information on the physiology and molecular network in ER bodies is available in specific reviews (Yamada et al., 2011; Nakano et al., 2014; Shirakawa and Hara-Nishimura, 2018).

Notably, ER bodies have been observed in only a few families of the order Brassicales, namely, Brassicaceae, Capparaceae, and Cleomaceae. Therefore, substrate(s) of the BGLUs could also be restricted to a narrow range of phylogenetic clades. Although PYK10, BGLU21, and BGLU22 neither have the amino acid signatures conserved in classical myrosinases nor display myrosinase activities toward aliphatic allyl GLS (sinigrin) *in vitro* (Ahn et al., 2010), previous studies on PEN2 led Nakano and co-authors to hypothesize that PYK10 can hydrolyze indole GLSs. As expected, root protein extracts of the *pyk10* and several ER-body mutants in *A. thaliana*, as well as the recombinant PYK10 protein, showed myrosinase activity against I3G (Nakano et al., 2017). Interestingly, co-expression analysis revealed that PYK10 is more closely related to biosynthetic genes for indole GLSs than to those of coumarin glucosides, putative substrates predicted from *in vitro* assays (Ahn et al., 2010; Nakano et al., 2017). Therefore, the physiological function of PYK10 *in planta* is more likely to be associated with GLS metabolism. Furthermore, a suite of informatics analyses performed in Nakano et al. (2017) indicated that a broad class of BGLUs, not limited to the classical myrosinases, may have the potential to hydrolyze GLSs, as described above.

The myrosinase activity of PYK10 toward indole GLSs is further supported by a very recent study on a new class of ER bodies, referred to as leaf ER bodies (Nakazaki et al., 2019). Compared with the aforementioned known classes of ER bodies, leaf ER bodies occur constitutively in a few types of epidermal cells in rosette leaves and accumulate both PYK10 and BGLU18. The *pyk10 bglu18* mutant was shown to lack leaf ER bodies and became more susceptible to attack by the terrestrial isopod *Armadillidium vulgare* compared with the wild type plants (Nakazaki et al., 2019). In addition, the levels of most endogenous GLS species decrease rapidly in homogenates of the rosette leaves, mainly due to the activities of TGG1 and TGG2, whereas degradation of 4-methoxyindol-3-ylmethyl glucosinolate (4MI3G) was found to be selectively and significantly delayed in the *pyk10 bglu18* mutant (Nakazaki et al., 2019). 4MI3G is a key GLS species in the PEN2-dependent immune pathway (Bednarek et al., 2009; Clay et al., 2009). Given that damage to leaves of the *tgg1 tgg2* mutant caused by *A. vulgare* in a feeding assay was comparable to that in the wild type, it has been suggested that leaf ER bodies are involved in the production of the defensive chemicals from 4MI3G that protect *A. thaliana* leaves against herbivore attack. Since neither *pyk10* nor *bglu18* single mutants were examined in these experiments, it would be of interest to determine whether BGLU18 can also hydrolyze indole GLSs. In combination with previous findings, these findings suggested that ER bodies can provide a further class of GLS–myrosinase compartment at the subcellular level (i.e., GLSs retained in vacuoles and myrosinases retained in the ER bodies), that plays an important role in plant defense against attacks of herbivores and pathogens.

## Do BGLU18 and BGLU33 Have Other Substrates in Addition to Glucose-Conjugated Abscisic Acid?

In addition to its potential roles in the ER bodies, BGLU18 is known to participate in abscisic acid (ABA) metabolism. ABA, one of the most important phytohormones active during a plant’s life cycle, is involved in a variety of biological processes, including the adaptation to environmental stresses (Sakata et al., 2014; Daszkowska-Golec, 2016). The cellular ABA level is partially regulated via a complex *de novo* biosynthetic pathway (Nambara and Marion-Poll, 2005). In addition, the discovery of ABA-glucosyltransferase, which generates an ABA glucosyl ester (ABA-GE), led us to hypothesize that release of ABA from the pool of inactive ABA analogs can potentially modulate ABA concentrations more dynamically (Xu et al., 2002). BG1/BGLU18 was reported as the first enzyme that can hydrolyze ABA-GE as part of the drought stress response (Lee et al., 2006). Subsequently, BG2/BGLU33 was identified as a further member of the ABA-GE hydrolases localized in vacuoles and was shown to play an important role under conditions of salt stress (Xu et al., 2012). Although the enzymatic potential of these BGLUs to hydrolyze ABA-GE and their relevance in ABA functions have been well investigated by independent groups (Merilo et al., 2015; Ondzighi-Assoume et al., 2016; Yamashita et al., 2016), it is questionable whether ABA-GE is the sole substrate of these BGLUs under physiological conditions, based on a consideration of the following observation.

In the first place, the response to drought and salinity is subject to complex regulation, not only by ABA but also by other small molecules including GLSs. Their contribution to the drought stress response has been discussed with regard to stomatal closure in guard cells, which is the major response of plants under low water conditions. The signal cascade for stomatal movement is initiated by multiple inputs from plant hormones and several primary/specialized metabolites in response to environmental stresses, whereas they are finally integrated into a single output — the production of reactive oxygen species (ROS) and Ca^2+^ oscillation followed by protein phosphorylation (Murata et al., 2015). Hence, depletion of one signal molecule could be compensated by the function of another compound. Notably, application of exogenous allyl ITC or several GLS breakdown products has been shown to induce stomatal closure in *A. thaliana* leaves (Khokon et al., 2011; Hossain et al., 2013). Furthermore, TGG1 and TGG2 have been reported to be major components of guard cells and shown to be involved in stomatal movement (Zhao et al., 2008; Islam et al., 2009). The stomatal closure induced by allyl ITC and the subsequent ROS production does not require endogenous ABA, but is dependent on methyl jasmonate (Khokon et al., 2011), indicating that ABA and ITC function as independent inputs for stomatal movement. Some researchers have hypothesized that the accumulation of TGGs and GLSs in guard cells represents the evolutionary origin of the GLS–myrosinase system, because stomata can serve as the initial gateway to bacterial invasion (Shirakawa and Hara-Nishimura, 2018). However, whether breakdown of internal GLSs in guard cells occurs under drought conditions to induce stomatal closure and the relevance of BGLU18 in this process are still to be investigated. Involvement of GLSs in the salt stress response has also been suggested based on the findings of several studies that have examined the fluctuation of GLS contents in Brassicaceae plants and the response of *A. thaliana* mutants lacking aliphatic GLSs under salinity stress (López-Berenguer et al., 2008; Keling and Zhu, 2010; Martinez-Ballesta et al., 2015), even though the detailed mechanisms remain unclear.

Secondly, the EE-type myrosinases tend to have a dual function as *S*- and *O*-glucosidases. PYK10 has been reported to hydrolyze coumarin glucosides such as scopolin *in vitro* (Ahn et al., 2010), whereas co-expression analysis has indicated that indole GLSs are more likely to be the actual substrates *in planta* (Nakano et al., 2017). PEN2 is also known to have enzymatic potential to catalyze the deglycosylation of 4-methylumbelliferyl-beta–D-*O*-glucoside, albeit at a lower rate than for the hydrolysis of indole GLSs (Bednarek et al., 2009). Drought stress appears to promote the degradation of a wide range of GLS species not limited to indole GLSs (Ren et al., 2009). One possible reason is that GLSs are hydrolyzed after transportation to the compartments/cells containing classical myrosinases. Alternatively, as is the case for GLS degradation induced by sulfur depletion or prolonged darkness (see the following sections), it is also conceivable that EE-type myrosinases, which may include BGLU18 and BGLU33, could hydrolyze other GLS subclasses.

Thirdly, whereas ABA is one of the indispensable hormone compounds in the plant kingdom, the BGLU subclass containing BGLU18 and BGLU33 is distributed only in the order Brassicales (Xu et al., 2004; Nakano et al., 2017). Notably, BGLU18 is a major and essential component of ER bodies, an organelle observed in only a few families of the order Brassicales, as described above. One possibility is that BGLU18 and BGLU33 may have other substrate(s) restricted to these small evolutionary clades, like PYK10. Another possible explanation is that there are several molecular systems to release ABA from the repository of inactive ABA derivatives, and these BGLUs may belong to one of those conserved only in the Brassicales. As a similar case, in some Brassicaceae plants, the metabolism of indole GLSs is closely related to that of auxin, another essential phytohormone (Malka and Cheng, 2017; Vik et al., 2018). Although similar machineries for dynamic regulation of ABA levels may exist in a wide range of plant families, such enzymes hydrolyzing inactive ABA derivatives have yet to be identified.

Taken together, the aforementioned findings indicate that we should not exclude the hypothesis that BGLU18 and BGLU33 can also function as myrosinases. If these BGLUs were proven to be dual-functional, the regulatory mechanism of these enzymatic activities *in planta* would inevitably attract greater attention.

## GLS Breakdown to Recycle Sulfur May Be Mediated by BGLU28 and BGLU30

This and the next section discuss the possibility that GLS itself could work as nutrient storage, not only as a precursor of toxic chemicals and signaling molecules. Brassicaceae plants containing GLSs need larger amounts of sulfur than other plants (Castro et al., 2003; Walker and Booth, 2003; Yang et al., 2006). Since GLSs have at least two sulfur atoms in each molecule and represent 10–30% of the total sulfur content in plant organs, these metabolites have been considered a potential source of sulfur for other metabolic processes (Falk et al., 2007). The effects of sulfur supply and depletion on GLS levels in plants have been well studied, including in agricultural practice (Falk et al., 2007; Schnug and Haneklaus, 2016). In *A. thaliana*, sulfur deficiency strongly induces down-regulation of GLS content as well as expression levels of GLS biosynthetic genes (Hirai et al., 2005; Zhang et al., 2011). To date, a few transcription factors, SULFUR LIMITATION 1 (SLIM1), SULFUR DEFICIENCY-INDUCED 1 (SDI1) and SDI2, have been reported to work as repressors of GLS biosynthesis under low sulfur conditions (Maruyama-Nakashita et al., 2006; Aarabi et al., 2016). In addition, developmental defects of GLS-less seeds as a result of mutations in GLS transporters (*gtr1 gtr2*) under sulfur deficiency further supported the potential role of GLSs as a sulfur reserve (Nour-Eldin et al., 2012). Nevertheless, most mechanisms underlying GLS turnover in that condition are still not known. It would be desirable to gain more direct evidence for the re-distribution of sulfur atoms from GLSs, e.g., by incorporating isotopes into primary sulfur metabolites from labeled GLSs.

Based on their observed up-regulation, BGLU28 and BGLU30 are suggested to be relevant in the hydrolysis of GLSs caused by sulfur depletion (Hirai et al., 2003, 2004; Maruyama-Nakashita et al., 2003; Nikiforova et al., 2003). BGLU28 and BGLU30 form a sister clade close to those containing PEN2 and PYK10 in the phylogenetic tree of *A. thaliana* BGLUs (Xu et al., 2004). Although their possible contribution to GLS turnover was suggested more than 15 years ago (Hirai and Saito, 2004), there have been few reports on the physiological functions of these genes. To our knowledge, only two studies (Zhang et al., 2014; Jackson et al., 2015) have monitored *BGLU28* promoter activity, using the GUS reporter gene as a marker of low-sulfur response upon plant hormone treatment and mutations in *SULTR1;2*. In this context, it should be noted that sulfur deficiency primarily affects contents of aliphatic GLSs, whereas PYK10 and PEN2 have been reported to hydrolyze only indole GLSs. If BGLU28 and BGLU30 can indeed hydrolyze aliphatic GLSs, the broad chemodiversity of EE-type myrosinases would undoubtedly gain more recognition. Compared with the TGGs that exhibit low selectivity for GLS species (Zhou et al., 2012), EE-type myrosinases may have narrower substrate specificities and thus regulate only particular biotic/abiotic stress responses. Both the protein functions and physiological roles of these BGLUs need to be further investigated beyond the context of sulfur assimilation.

## Activation of GLS Turnover in Darkness and Possible Contribution of BGLU30 to Recover Carbohydrates

Light is an essential energy source for the development and metabolism of higher plants, and the expression levels of a number of genes are known to be regulated by light; for example, the sulfur assimilation pathway is activated during the light period (Kocsy et al., 1997; Kopriva et al., 1999; Pruneda-Paz and Kay, 2010). Although the photo-regulation of sulfur assimilation has been investigated in different species under a variety of growth conditions (Buwalda et al., 1988; Passera et al., 1989; Lee E.-J. et al., 2007; Lee et al., 2011), there tends to be little consensus regarding its coordination with GLS biosynthesis/catabolism.

The regulation of GLS metabolism by light has been discussed from two aspects, namely, the substantial degradation of GLSs under conditions of prolonged darkness and the diurnal fluctuation of GLS contents. In *A. thaliana*, there is a marked reduction in the GLS content of leaves under conditions of extended darkness (Huseby et al., 2013; Brandt et al., 2018). Given that inhibition of photosynthesis is a strong stimulus inducing carbohydrate starvation and leaf senescence, GLS degradation may be a mechanism designed to cope with nutrient starvation via the mobilization of D-glucose units from those molecules. In this regard, *BGLU30*, also referred to as *DARK-INDUCIBLE 2* (*DIN2*) or *SENESCENCE-RELATED GENE 2* (*SRG2*), is known to be significantly up-regulated in response to prolonged darkness, senescence, and sugar starvation (Fujiki et al., 2001; Lee J. et al., 2007). The key factor for the induction of *BGLU30* expression appears to be endogenous sugar levels rather than light conditions. A high abundance of *BGLU30* transcript was detected in detached leaves even under illumination in the presence of a photosynthesis inhibitor, 3-(3,4-dichlorophenyl)-1,1-dimethylurea, whereas it was barely detected under co-treatment with sucrose (Fujiki et al., 2001). Given that the *BGLU30* expression is also induced by sulfur depletion, this enzyme may regulate a release of stored GLSs to overcome nutrient starvation under various conditions. Notably, the expression of *BGLU28* is induced neither by prolonged darkness nor by sugar starvation. In addition to their enzymatic functions, the difference in the regulatory systems between these closely related BGLUs is of particular interest.

Cooperating with the sulfur assimilation pathways, GLS levels have been shown to be higher during the day than at night in *A. thaliana* (Huseby et al., 2013). Simultaneously, the expression levels of GLS biosynthetic genes as well as the incorporation of inorganic ^35^S into GLSs were found to be enhanced by light. Moreover, a further study revealed a diurnal increase in total myrosinase activity and the abundance of TGG1 and TGG2 proteins in *A. thaliana* seedlings (Brandt et al., 2018). These findings accordingly indicate that GLS metabolism is highly co-regulated with sulfur assimilation in response to the circadian rhythm, even though the physiological importance of this phenomenon remains unclear. Since the correlation between GLS contents and the expression levels of GLS biosynthetic genes was relatively low during the light period (Rosa, 1997; Klein et al., 2006; Schuster et al., 2006), not only *de novo* biosynthesis but also turnover could regulate the diurnal rhythm of endogenous GLSs. In this context, however, a contribution of BGLU30 to GLS degradation is less likely because an increase of *BGLU30* transcripts was observed only after 12 h or longer of dark treatment (Fujiki et al., 2001; Lee J. et al., 2007). In addition to the transcriptomic changes of other *BGLUs*, post-translational regulation of myrosinase activities, including those of TGG1 and TGG2 (Brandt et al., 2018), should be considered in order to gain a better understanding of the dynamic control of GLS contents over a 24-h period.

## Additional Roles of Classical TGGs Beyond the “Mustard Oil Bomb”

Several new findings on members of the QE-type myrosinases (TGG1-TGG6) have indicated their broader physiological importance in various situations, beyond the classical intercellular “mustard oil bomb” system deployed against predators. As described above, TGG1 and TGG2 might be involved in guard cell ITC production (Zhao et al., 2008; Islam et al., 2009) and the diurnal control of GLS levels in the absence of tissue disruption (Brandt et al., 2018). In this section we discuss two recent studies that have reported the detailed analysis of root-specific TGGs and the function of TGG6, which had hitherto been considered a pseudogene (Fu et al., 2016a,b).

Currently, QE-type myrosinases are classified into two subclasses, namely, Myr I and Myr II (Wang et al., 2009a). Members of subclass Myr I are found in all GLS-containing plants and are typically deposited in myrosin cells, thereby establishing the compartmentalization required for the intercellular GLS–myrosinase system (Rask et al., 2000). Thus, Myr I class myrosinases are considered to be critical for biochemical defense against herbivores. Myr II members differ from those in subclass Myr I with respect to several features, including sequence divergence and gene structure (Wang et al., 2009a; Nong et al., 2010; Fu et al., 2016a). Functional analysis of TGG4 and TGG5, the first examples of the Myr II subfamily to be examined, indicated their root-specific roles differ from those of leaf-localized Myr I members. A comparison of the enzymatic properties of TGG4 and TGG5 with those of TGG1 using recombinant proteins expressed in *Pichia pastoris* revealed that TGG4 and TGG5 have higher stability than TGG1 under adverse conditions, such as high temperature, low pH, and excess NaCl (Andersson et al., 2009). In a more recent study, Fu et al. investigated the tissue localization, regulation of root growth, and possible contribution to auxin biosynthesis of TGG4 and TGG5 (Fu et al., 2016b). Analyses of GUS reporter gene expression and myrosinase activities of the single and double KO mutants tended to indicate that TGG5 is more predominant in roots. The defective root elongation under flooded conditions and expression patterns of the auxin-responsive *DR5:GUS* reporter system in these mutants indicated that TGG4 and TGG5 may contribute to auxin biosynthesis at the root tip by hydrolyzing indole GLSs to form indole-3-acetonitrile, a direct precursor of indole-3-acetic acid, even though their enzymatic activities against indole GLSs remain to be confirmed. Given that the aforementioned experiments were performed under non-invasive conditions, it is conceivable that GLS breakdown by the Myr II myrosinases may be less dependent on tissue damage. Hence, not only EE-type but also classical myrosinases could regulate subcellular GLS–myrosinase systems in addition to the so-called “mustard oil bomb.” Our understanding in this regard will be ameliorateded by single-cell-level analysis of the specialized compartments in different cell types, as reviewed by Chen et al. (2019) in this issue.

A further surprising finding is that *TGG6*, which had previously been reported to be a pseudogene but is specifically expressed in pollen (Wang et al., 2009b), is still functional in a number of *A. thaliana* accessions (Fu et al., 2016a). The authors identified 10 functional alleles of *TGG6* from 29 accessions and the recombinant TGG6 derived from Tsu-1 showed a clear myrosinase activity against sinigrin. The predominant expression pattern of functional *TGG6* alleles in pollen was relatively similar to that of the non-functional *TGG6* in Col-0. Given that an ortholog of *TGG6* is predicted to be functional in *Arabidopsis lyrata*, an outcrossing relative of *A. thaliana* (Kusaba et al., 2001; Sherman-Broyles et al., 2007; Tang et al., 2007), it is suggested that its ancestral role was the defense of pollen against herbivores. However, subsequent evolutionary acquisition of a self-fertilization system rendered it no longer critical in *A. thaliana*, thereby resulting in a loss of function in most accessions. A hypothesis proposed based on the findings on TGG6 is that *BGLUs* with low expression levels in Col-0, the most studied accession of *A. thaliana*, could still be functional in other accessions. As GLS compositions have become significantly differentiated during evolution even within the same species (Kliebenstein et al., 2001; Edger et al., 2015; Kliebenstein and Cacho, 2016; Barco and Clay, 2019), it is possible that a BGLU plays a critical role in a few accessions but is not important in others. Accession- and species-wide analysis of the same BGLU orthologs may help us to gain a better understanding of the specific functions of these enzymes *in planta* and how they have acquired these specific roles during the course of evolution.

## Current Understanding of the Other Brassicales-Specific BGLU

Other than BGLUs described above, current insights on the Brassicales-specific BGLUs are highly limited. To get an overview of BGLU18–BGLU33, we performed a public data analysis using ePlant^1^ (Waese et al., 2017) (Figure 2). In many cases, only a few publications were extracted when each BGLU was used as a query (Figure 2A). Our lack of knowledge on several BGLUs is probably due to their almost undetectable expression. Using the Plant eFP viewer, we can see that signal levels of these *BGLUs* in the Affymetrix ATH1 microarray are very low in almost all tissues, except for *BGLU18* and *PEN2* (Figure 2B). Although the low signal does not mean a low abundance of the actual transcript, it hinders performance of many biological analyses. Instead, some *BGLUs* exhibit very specific expression patterns in particular tissue(s), e.g., *BGLU19* in mature seeds (Figure 2B and Supplementary Table S1). Moreover, expression levels of each *BGLU* under broad abiotic stresses are summarized (Figure 2C and Supplementary Tables S2, S3). In addition to the known information such as up-regulation of *BGLU18* by drought or wounding, we can expect drastic changes in the expression of uncharacterized *BGLUs* in response to specific stresses, such as the highly increased expression of *BGLU24* in roots as a result of osmotic and salt stress. It should also be noted that according to ATTED-II^2^ (Obayashi et al., 2018), several *BGLUs* show high co-expression scores with specifier proteins: *BGLU19* with *NSP2*, *BGLU30* with *NSP5*, and *PYK10* with *NSP1/NSP3/NSP4* (crosshybridized to the same probe). Broad end products might be generated even in the atypical myrosinase-mediated GLS breakdown. Hence, numerous public data previously collected could help us hypothesize a specific relevance of these BGLU(s) in particular developmental stages or abiotic stress responses.

## Concluding Remarks and Future Perspective

In the past decade, subsequent to the identification of PEN2 in 2009, only PYK10 has been reported as a further member of the EE-type myrosinases. As reviewed here, however, this does not exclude the possible contribution of Brassicales-specific BGLUs to GLS breakdown under specific conditions. In particular, discovery of EE-type myrosinases catalyzing the hydrolysis of aliphatic GLSs in addition to indole GLSs would generate heightened interest amongst researchers in their physiological importance and catalytic mechanisms, compared with the classical myrosinases. In addition, recent studies have demonstrated that classical myrosinases such as TGGs can also participate in non-tissue-disruptive GLS breakdown beyond the well-known “mustard oil bomb” system at the tissue level. Furthermore, we should pay attention to the accession-wide functional differentiation of the same ortholog with a single species, as highlighted in the case of TGG6. For example, substrate specificity may be dependent on the GLS composition of an accession. It may also be possible that a BGLU has evolved to regulate specialized signals initiated by GLS species present in only a few accessions. Addressing the broad distribution and different myrosinase activities of BGLUs in Brassicaceae and closely related families will enable us to elucidate how the multi-functionality of GLSs is controlled *in planta*, and how the GLS–myrosinase systems have diversified during evolution.

*In vitro* enzymatic assays using recombinant proteins would be helpful in determining the physiological functions of these enzymes *in planta*. In this regard, *Pichia pastoris* and tobacco BY-2 cells seem to be the preferable organisms to express the *A. thaliana* myrosinases with enzymatic functions, according to previous studies (Andersson et al., 2009; Bednarek et al., 2009; Fu et al., 2016a; Nakano et al., 2017). However, we should bear in mind the fact that the myrosinase assay using sinigrin, an easily available substrate, may not identify (and perhaps has not identified) the actual enzymatic potential of candidate BGLUs of interest. As the classical myrosinases tend to hydrolyze a diverse range of GLS structures (Zhou et al., 2012), most studies have examined the “myrosinase activity” using only sinigrin, a GLS with a simple allyl chain. However, the EE-type myrosinases may have a restricted substrate selectivity and require optimal conditions to work under particular conditions, as emphasized by the findings for PEN2 and PYK10, which preferentially hydrolyze indole GLSs within a narrow optimal pH range (Bednarek et al., 2009; Nakano et al., 2017). It is also notable that sinigrin is detected only in certain *A. thaliana* accessions other than Col-0 (Kliebenstein et al., 2001). Since the side chain structures of GLSs can substantially alter the physicochemical properties of the corresponding degradation products such as ITCs, it would be preferable to examine the activity of myrosinases against a broader range of GLS species to establish the physiological importance of the BGLUs of interest. Recent advances in the methods for extraction of intact GLSs from plant materials and quantitative analysis of GLS contents may contribute to promoting this approach (Bianco et al., 2017; Doheny-Adams et al., 2017).

In addition to the abiotic stresses discussed herein, there are a few physiological conditions that are potentially related to GLS metabolism and catabolism (Table 2). For example, pre-treatment with phenethyl ITC confers heat stress tolerance on *A. thaliana* seedlings, probably by up-regulating a suite of heat-shock proteins (Hara et al., 2012; Kissen et al., 2016). In addition, the mechanisms underlying the degradation of total GLS amount independent of TGG1 and TGG2 during early developmental stages remain to be clarified (Barth and Jander, 2006). Given that up-regulation of particular BGLUs has yet to be observed under these conditions, we should consider the post-translational regulation of myrosinase activities with regard to myrosinase-associated proteins or small molecule elicitors such as ascorbate (Wittstock et al., 2016a; Bhat and Vyas, 2019; Chen et al., 2019). Under non-disruptive conditions, the physiological functions of myrosinases, including TGGs, are probably controlled more strictly and dynamically than expected till now.

## Author Contributions

RS and MH prepared the manuscript. RS prepared the figures and tables. MH finalized the manuscript for submission.

## Conflict of Interest Statement

The authors declare that the research was conducted in the absence of any commercial or financial relationships that could be construed as a potential conflict of interest.

## Footnotes

1 https://bar.utoronto.ca/eplant/

2 http://atted.jp
