# Supplementary material for: Atypical Myrosinase as a Mediator of Glucosinolate Functions in Plants
Source: Front Plant Sci. 2019 Aug 6;10:1008. doi: 10.3389/fpls.2019.01008 (PMC6691170; doi:10.3389/fpls.2019.01008)
Supplement: Supplementary file 1 [file Data_Sheet_1.PDF]

Supplementary Table S1. Top three tissues exhibiting the highest signal levels of *BGLU18–BGLU33*.

Gene name	AGI ID	1st		2nd		3rd		median[b]
		tissue	level[a]	tissue	level[a]	tissue	level[a]	
BGLU18/BG1	At1g52400	leaf 7, petiol	3647	flowers stage 15, carpels	3368	veg rosette (for phase change)	3360	1554
BGLU19	At3g21370	seeds, stage 10, w/o siliques	6122	seeds, stage 9, w/o siliques	4956	seeds, stage 8, w/o siliques	2113	1
BGLU20	At1g75940	flowers stage 10/11	2247	flowers stage 12	929	flowers stage 12, stamens	812	1
BGLU21	At1g66270	Roots, 7 days	1934	Roots, 17 days	1817	seed, 24h water	183	4
BGLU22	At1g66280	Roots, 7 days	1934	Roots, 17 days	1817	seed, 24h water	183	4
BGLU23/PYK10	At3g09260	hypocotyl	2983	Roots, 7 days	2982	Roots, 17 days	2399	14
BGLU24	At5g28510	seeds, stage 10, w/o siliques	14	Roots, 7 days	12	seeds, stage 9, w/o siliques	10	3
BGLU25	At3g03640	flowers stage 12, sepals	302	flowers stage 12, petals	262	flowers stage 15, sepals	255	108
BGLU26/PEN2	At2g44490	leaf 7, proximal half	1209	leaf 7, distal half	1200	senescing leaves	1084	310
BGLU27	At3g60120	flowers stage 15, sepals	32	senescing leaves	18	hypocotyl	15	7
BGLU28	At2g44460	rosette leaf # 2	201	siliques, w/ seeds stage 3	141	flowers stage 15, carpels	81	10
BGLU29	At2g44470	seeds, stage 7, w/o siliques	860	seeds, stage 6, w/o siliques	533	siliques, w/ seeds stage 5	201	6
BGLU30	At3g60140	senescing leaves	286	flowers stage 15, sepals	212	siliques, w/ seeds stage 3	207	7
BGLU31	At5g24540	senescing leaves	45	flowers stage 15	17	flowers stage 15, pedicels	15	3
BGLU32	At5g24550	seeds, stage 10, w/o siliques	6	flowers stage 15, stamen	6	flowers stage 15, sepals	5	3
BGLU33/BG2	At2g32860	leaf 7, petiol	416	1st node	222	cauline leaves	216	49

The Affymetrix ATH1 array data was extracted from the AtGenExpress eFP viewer in ePlant (https://bar.utoronto.ca/eplant/) (Waese et al., 2017). Original data comes from Schmid et al. (2005) and Nakabayashi et al. (2005).

[a] Average signal levels of two or three replicates are shown.

[b] Median of signals from total 140 samples.

Supplementary Table S2. Transcriptional changes of *BGLU18–BGLU33* in shoots under abiotic stresses.

Gene name	AGI ID	signal levels of control[a]		relative expressions (log_2[Fold-Change])							
				cold		osmotic		salt		drought	
		6 h	24 h	6 h	24 h	6 h	24 h	6 h	24 h	6 h	24 h
BGLU18/BG1	At1g52400	1409	1167	-0.6	-0.1	0.5	**-1.4**	0.3	0.1	**1.4**	**1.3**
BGLU19	At3g21370	7	4	**-2.2**	**-1.2**	**-2.1**	**-1.6**	**-2.0**	**-1.7**	**-2.0**	**-1.2**
BGLU20	At1g75940	2	1	**-1.6**	-0.1	**-2.1**	0.3	1.0	-0.8	**-2.1**	**-1.4**
BGLU21	At1g66270	8	5	-0.3	**-3.5**	**-3.7**	-0.1	**-2.5**	**-2.6**	0.3	0.4
BGLU22	At1g66280	8	5	-0.3	**-3.5**	**-3.7**	-0.1	**-2.5**	**-2.6**	0.3	0.4
BGLU23/PYK10	At3g09260	925	698	-0.8	-0.7	**-1.8**	-1.0	-0.8	**-1.6**	1.0	0.5
BGLU24	At5g28510	3	5	-0.3	**-1.2**	0.2	0.4	0.5	**-1.2**	0.6	-0.8
BGLU25	At3g03640	64	43	0.9	**4.3**	0.7	0.9	1.0	**1.6**	0.4	0.5
BGLU26/PEN2	At2g44490	897	375	0.8	**2.0**	0.1	0.9	-0.4	-0.2	-0.3	0.4
BGLU27	At3g60120	9	8	0.1	0.0	0.2	**1.6**	-0.7	-0.3	-0.5	-0.4
BGLU28	At2g44460	11	11	0.4	**-1.1**	**-1.0**	**-1.6**	0.4	0.1	-0.4	0.9
BGLU29	At2g44470	5	6	0.7	**-1.1**	0.0	-0.3	0.7	-0.5	0.3	0.3
BGLU30	At3g60140	3	8	**1.1**	-0.8	**2.3**	**5.8**	**2.6**	-0.4	**1.3**	-0.4
BGLU31	At5g24540	8	2	-0.9	0.7	-0.6	**1.0**	-1.0	0.6	**-1.8**	**1.8**
BGLU32	At5g24550	4	2	**-2.1**	**1.3**	**-1.0**	0.2	0.0	**1.0**	**-3.0**	**1.7**
BGLU33/BG2	At2g32860	174	181	-0.1	0.1	0.2	-0.2	0.1	0.0	0.2	0.3

The Affymetrix ATH1 array data was extracted from the Abiotic Stress eFP viewer in ePlant (https://bar.utoronto.ca/eplant/) (Waese et al., 2017). Original data comes from Kilian et al. (2007).

[a] Average signal levels of two replicates are shown.

Supplementary Table S2 (continued).

Gene name	AGI ID	relative expressions (log2[Fold-Change])									
		genotoxic		oxidative		UV-B		wounding		heat	
		6 h	24 h	6 h	24 h	6 h	24 h	6 h	24 h	6 h	24 h
BGLU18/BG1	At1g52400	0.0	0.1	0.3	0.0	0.3	-0.5	**1.3**	**1.3**	**-1.8**	-0.5
BGLU19	At3g21370	-0.8	**-2.1**	**-2.3**	**-1.1**	**-2.4**	**-1.0**	**1.0**	0.1	**-2.4**	**1.2**
BGLU20	At1g75940	-0.5	-0.3	0.1	**-1.3**	0.6	-0.4	-0.4	-0.9	0.2	0.3
BGLU21	At1g66270	**-1.3**	-0.8	-0.2	**2.0**	**-1.3**	**-3.4**	**2.8**	**2.2**	-0.2	0.3
BGLU22	At1g66280	**-1.3**	-0.8	-0.2	**2.0**	**-1.3**	**-3.4**	**2.8**	**2.2**	-0.2	0.3
BGLU23/PYK10	At3g09260	-0.4	-0.6	-0.7	-0.1	-0.8	**-1.1**	0.7	**1.2**	**-1.7**	0.2
BGLU24	At5g28510	-0.2	-0.7	-0.6	-0.2	**-1.7**	-0.5	**1.1**	0.4	**-1.9**	-0.2
BGLU25	At3g03640	0.2	0.4	0.1	0.6	-0.2	0.7	0.8	0.3	0.5	-0.1
BGLU26/PEN2	At2g44490	0.0	0.3	-0.3	0.4	0.3	0.1	-0.2	0.6	-0.1	0.2
BGLU27	At3g60120	**-3.4**	**-3.2**	-1.0	0.5	0.5	-0.8	-0.4	0.4	0.1	0.2
BGLU28	At2g44460	0.4	0.6	0.3	**3.3**	1.0	0.0	0.1	0.3	0.0	0.1
BGLU29	At2g44470	-0.6	-0.4	-0.2	-0.4	-0.6	-0.4	0.4	0.8	0.4	0.2
BGLU30	At3g60140	0.8	-0.2	0.6	**4.5**	**2.9**	0.0	**4.8**	**2.0**	**1.3**	-0.5
BGLU31	At5g24540	**-1.7**	-0.1	**-1.8**	**1.4**	**1.1**	**1.1**	**-3.1**	**1.8**	-0.6	1.0
BGLU32	At5g24550	**-2.1**	**1.3**	**-1.1**	0.1	**-2.1**	0.8	0.4	**1.8**	**-1.4**	0.8
BGLU33/BG2	At2g32860	0.3	0.5	0.3	0.4	-0.9	-0.4	-0.2	-0.1	**-1.5**	0.1

Supplementary Table S3. Transcriptional changes of *BGLU18–BGLU33* in roots under abiotic stresses.

Gene name	AGI ID	signal levels of control[a]		relative expressions (log_2[Fold-Change])							
				cold		osmotic		salt		drought	
		6 h	24 h	6 h	24 h	6 h	24 h	6 h	24 h	6 h	24 h
BGLU18/BG1	At1g52400	9	10	**1.7**	0.7	1.0	**2.8**	0.6	**2.0**	**1.3**	0.1
BGLU19	At3g21370	3	2	-0.8	0.3	0.2	**1.4**	0.5	0.9	0.5	-0.3
BGLU20	At1g75940	4	1	-0.7	**2.2**	**-2.1**	**1.7**	**-1.4**	0.6	-1.0	**1.5**
BGLU21	At1g66270	1711	1665	-0.2	0.0	-0.5	-0.1	-0.6	-0.7	0.2	0.6
BGLU22	At1g66280	1711	1665	-0.2	0.0	-0.5	-0.1	-0.6	-0.7	0.2	0.6
BGLU23/PYK10	At3g09260	3505	3246	0.0	0.2	-0.2	0.2	0.0	0.2	0.1	0.0
BGLU24	At5g28510	8	6	0.7	0.6	**5.5**	**7.1**	**5.7**	**6.1**	**3.2**	**1.1**
BGLU25	At3g03640	110	106	0.1	**3.0**	**1.1**	0.6	0.4	0.7	0.7	0.1
BGLU26/PEN2	At2g44490	592	507	0.4	**1.3**	-0.2	0.1	**1.5**	**1.0**	-0.1	-0.2
BGLU27	At3g60120	10	20	**2.2**	**2.0**	**1.8**	**1.3**	**6.7**	**4.5**	**1.5**	-0.6
BGLU28	At2g44460	6	5	0.2	0.9	0.0	**1.2**	**1.9**	0.7	-0.1	**-1.3**
BGLU29	At2g44470	4	6	0.6	0.0	0.9	0.5	0.9	0.1	0.5	-0.3
BGLU30	At3g60140	84	109	**1.4**	**1.2**	**4.3**	**4.5**	**3.3**	**3.5**	**2.1**	0.8
BGLU31	At5g24540	6	7	**1.6**	**1.4**	**1.1**	-0.4	**5.1**	**3.4**	-0.3	-0.5
BGLU32	At5g24550	9	16	0.9	**1.2**	0.7	-0.9	**5.2**	-0.5	-1.0	-0.9
BGLU33/BG2	At2g32860	6	5	0.4	0.0	0.4	**1.1**	0.1	**1.3**	-0.7	0.3

The Affymetrix ATH1 array data was extracted from the Abiotic Stress eFP viewer in ePlant (https://bar.utoronto.ca/eplant/) (Waese et al., 2017). Original data comes from Kilian et al. (2007).

[a] Average signal levels of two replicates are shown.

Supplementary Table S3 (continued).

Gene name	AGI ID	relative expressions (log2[Fold-Change])									
		genotoxic		oxidative		UV-B		wounding		heat	
		6 h	24 h	6 h	24 h	6 h	24 h	6 h	24 h	6 h	24 h
BGLU18/BG1	At1g52400	**1.6**	0.9	-0.6	0.4	0.4	0.8	**2.4**	**1.7**	-0.4	-0.1
BGLU19	At3g21370	0.9	-0.8	-0.2	-0.8	-0.1	-0.7	**-1.7**	-0.5	0.9	**1.1**
BGLU20	At1g75940	0.5	0.9	**-2.2**	**1.2**	-0.9	1.0	-0.2	0.2	**-1.2**	**-1.3**
BGLU21	At1g66270	-0.2	-0.5	-0.2	-0.3	-0.2	0.0	0.2	0.3	-0.9	0.4
BGLU22	At1g66280	-0.2	-0.5	-0.2	-0.3	-0.2	0.0	0.2	0.3	-0.9	0.4
BGLU23/PYK10	At3g09260	-0.2	0.0	-0.2	0.0	-0.1	0.1	0.0	0.1	-0.2	-0.1
BGLU24	At5g28510	-0.3	0.9	0.3	**2.4**	-1.0	0.6	0.5	**1.9**	0.4	0.6
BGLU25	At3g03640	0.3	0.2	-0.3	0.2	-0.1	-0.1	0.0	0.1	0.3	0.3
BGLU26/PEN2	At2g44490	-0.3	0.2	0.0	-0.3	0.0	0.3	0.0	0.2	0.2	0.0
BGLU27	At3g60120	**1.2**	-0.3	0.1	-0.5	-0.7	0.5	0.3	-0.4	0.3	-0.1
BGLU28	At2g44460	-0.6	0.5	-0.2	**2.7**	-0.5	-0.5	0.2	-0.2	0.4	0.7
BGLU29	At2g44470	0.8	0.4	0.1	0.2	0.5	0.1	0.6	0.3	0.1	0.6
BGLU30	At3g60140	**1.7**	**1.2**	0.7	**1.7**	0.5	**1.3**	**1.3**	**1.3**	**1.5**	0.6
BGLU31	At5g24540	**1.3**	0.0	-0.1	-0.2	-0.4	0.3	0.7	0.1	0.1	0.0
BGLU32	At5g24550	0.9	**-1.1**	-0.6	**-1.9**	**-2.3**	**-1.2**	-0.4	-0.8	0.0	-0.6
BGLU33/BG2	At2g32860	0.4	**1.2**	**-1.2**	0.6	-0.6	0.1	0.0	0.4	**-1.2**	0.6

References

1. Kilian, J., Whitehead, D., Horak, J., Wanke, D., Weinl, S., Batistic, O., et al. (2007) The AtGenExpress global stress expression data set: protocols, evaluation and model data analysis of UV-B light, drought and cold stress responses. *Plant J* 50, 347–363.
2. Nakabayashi, K., Okamoto, M., Koshiba, T., Kamiya, Y., and Nambara, E. (2005) Genome-wide profiling of stored mRNA in Arabidopsis thaliana seed germination: epigenetic and genetic regulation of transcription in seed. *Plant J* 41, 697-709.
3. Schmid, M., Davison, T. S., Henz, S. R., Pape, U. J., Demar, M., Vingron, M., et al. (2005) A gene expression map of Arabidopsis thaliana development. *Nat Genet* 37, 501-506.
4. Waese, J., Fan, J., Pasha, A., Yu, H., Fucile, G., Ruian Shi, R., et al. (2017) ePlant: visualizing and exploring multiple levels of data for hypothesis generation in plant biology. *Plant Cell* 29, 1806–1821.
